# Refractory mastoiditis as the initial manifestation of granulomatosis with polyangiitis:a case report and literature review

**DOI:** 10.3389/fimmu.2026.1753797

**Published:** 2026-06-29

**Authors:** Xinying Cui, Chunchao Wang, Jingchun Jin

**Affiliations:** Department of Rheumatology, the Affiliated Hospital of Yanbian University (Yanbian Hospital), Yanji, China

**Keywords:** antineutrophil cytoplasmic antibodies, case report, granulomatosis with polyangiitis, mastoiditis, refractory otitis media, rituximab

## Abstract

**Background:**

Granulomatosis with Polyangiitis (GPA) is an antineutrophil cytoplasmic antibody (ANCA)-associated vasculitis that predominantly affects the upper or lower respiratory tracts and the kidney. While otologic involvement is relatively common in GPA, isolated destructive mastoiditis as the initial manifestation is exceedingly rare, often leading to misdiagnosis.

**Case presentation:**

A 69-year-old female presented with a 3-months history of progressive bilateral hearing loss and otorrhea. Based on clinical and radiological findings, she was initially diagnosed with acute mastoiditis but showed no response to antibiotic therapy. She subsequently underwent right sided canal-wall-up mastoidectomy; however, postoperative symptoms persisted, and pathological examination only revealed inflammatory granulation tissue. Her condition further deteriorated, with the development of contralateral ear involvement and persistent low-grade fever. Serological testing eventually showed a proteinase 3 antineutrophil cytoplasmic antibodies (PR3-ANCA) level>200 RU/mL and chest computed tomography (CT) demonstrated multiple bilateral pulmonary nodules. The patient was thus diagnosed with GPA and initiated on methylprednisolone pulse therapy, followed by rituximab (RTX).

**Conclusion:**

This case highlights that, in patients with refractory mastoiditis unresponsive to conventional antibiotics and surgical intervention, otolaryngologists should maintain a high index of suspicion for underlying systemic vasculitides and initiate early ANCA testing. Such measures are critical to avoid diagnostic delays and prevent irreversible organ damage.

## Introduction

Granulomatosis with Polyangiitis (GPA) is a systemic necrotizing granulomatous vasculitis closely associated with antineutrophil cytoplasmic antibodies (ANCA), primarily affecting small-to-medium-sized blood vessels. About 80%-95% of the patients the first symptoms of GPA are otorhinolaryngological manifestations of head and neck including nose/sinuses, ears, eyes, larynx or trachea, oral cavity and salivary glands (1). While a subset of patients may present with the classic triad of GPA involvement of the upper respiratory tract, lungs, and kidneys (2, 3). Otitis media, a common middle ear manifestation of GPA, occurs in approximately 20%-40% of cases (4). However, these otologic symptoms are typically secondary to nasopharyngeal inflammation and Eustachian tube dysfunction or accompany other systemic manifestations of GPA (5); in contrast, isolated and destructive mastoiditis as the initial presentation of GPA is exceedingly rare, often leading to misdiagnosis as an infectious disease (6).

Here, we present the case of a patient with rare manifestation of GPA (isolated destructive mastoiditis as the initial presentation) and after initiating RTX, the ear symptoms and bilateral pulmonary nodules of the patients were obviously improved, coinciding with a reduction in PR3-ANCA titers. This report aims to enhance the recognition of atypical presentations of AAVs and improve the diagnosis of GPA. In particular, early reports describing cases with isolated organ involvement may help deepen understanding of the disease and reduce both misdiagnosis and missed diagnosis.

## Case presentation

A 69-year-old female was admitted to the Department of Rheumatology and Immunology on October 8, 2024, due to progressive bilateral hearing loss (3 months’ duration) with sudden worsening on October 1, 2024.

### Clinical course prior to admission

**Figure 1:**
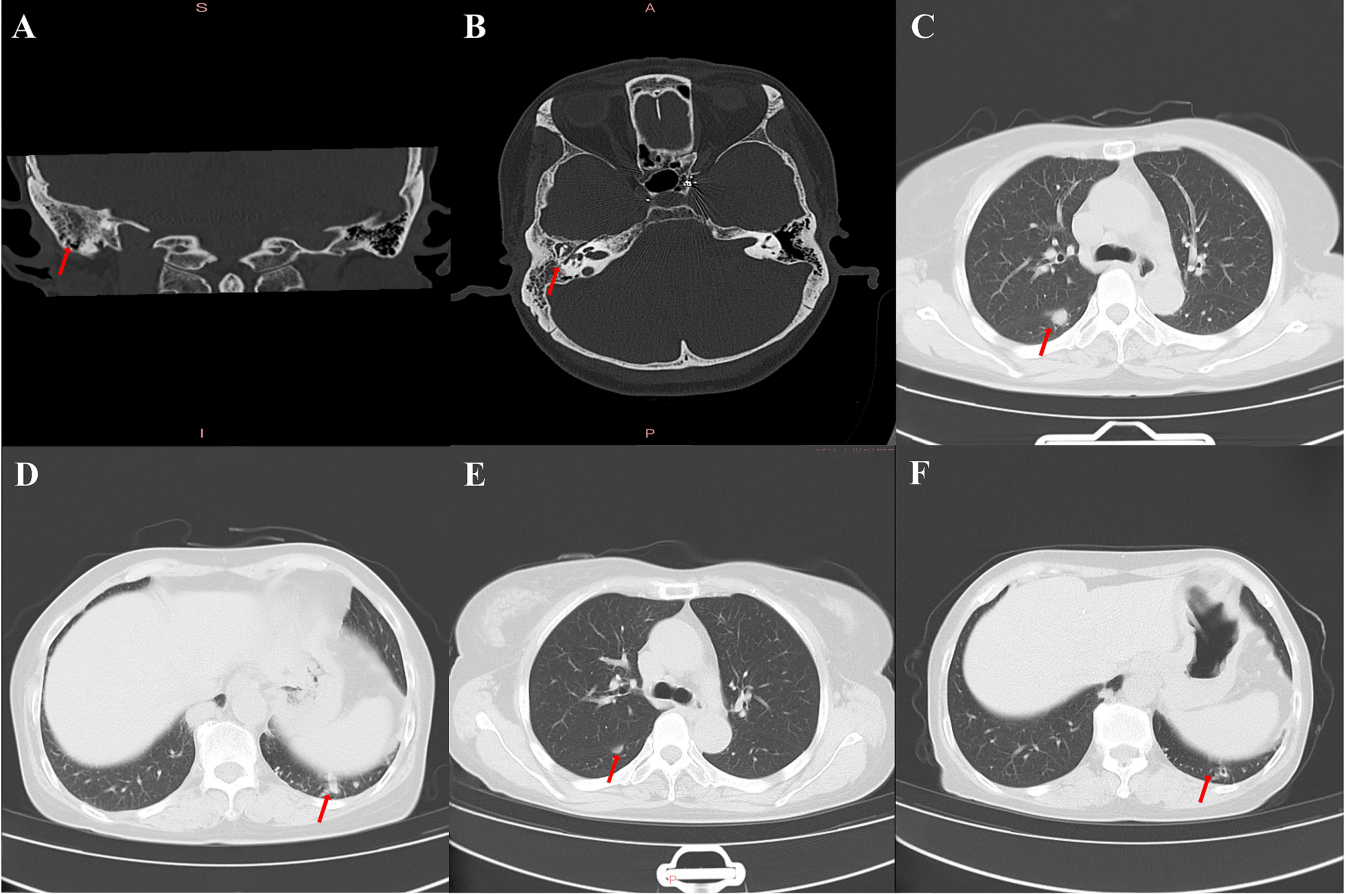
Imaging manifestations. **(A, B)** Right mastoid sclerosis, loss of air cells, and soft-tissue density in the middle ear and mastoid cavity. **(C, D)** Multiple bilateral solid pulmonary nodules. **(E, F)** After treatment significant reduction in the size of bilateral pulmonary nodules.

In July 2024, the patient developed an unprovoked dull ache in the right ear (not limited to the ear canal), accompanied by hearing loss (**Supplementary Figure 1A**), intermittent low-pitched tinnitus and otorrhea. She underwent temporal bone computed tomography (CT), which showed right mastoid sclerosis, loss of air cells, and soft-tissue density in the middle ear and mastoid cavity (**Figures 1A, B**). Despite a 12-day course of broad-spectrum cephalosporins, no symptomatic improvement was observed.

**Figure 2:**
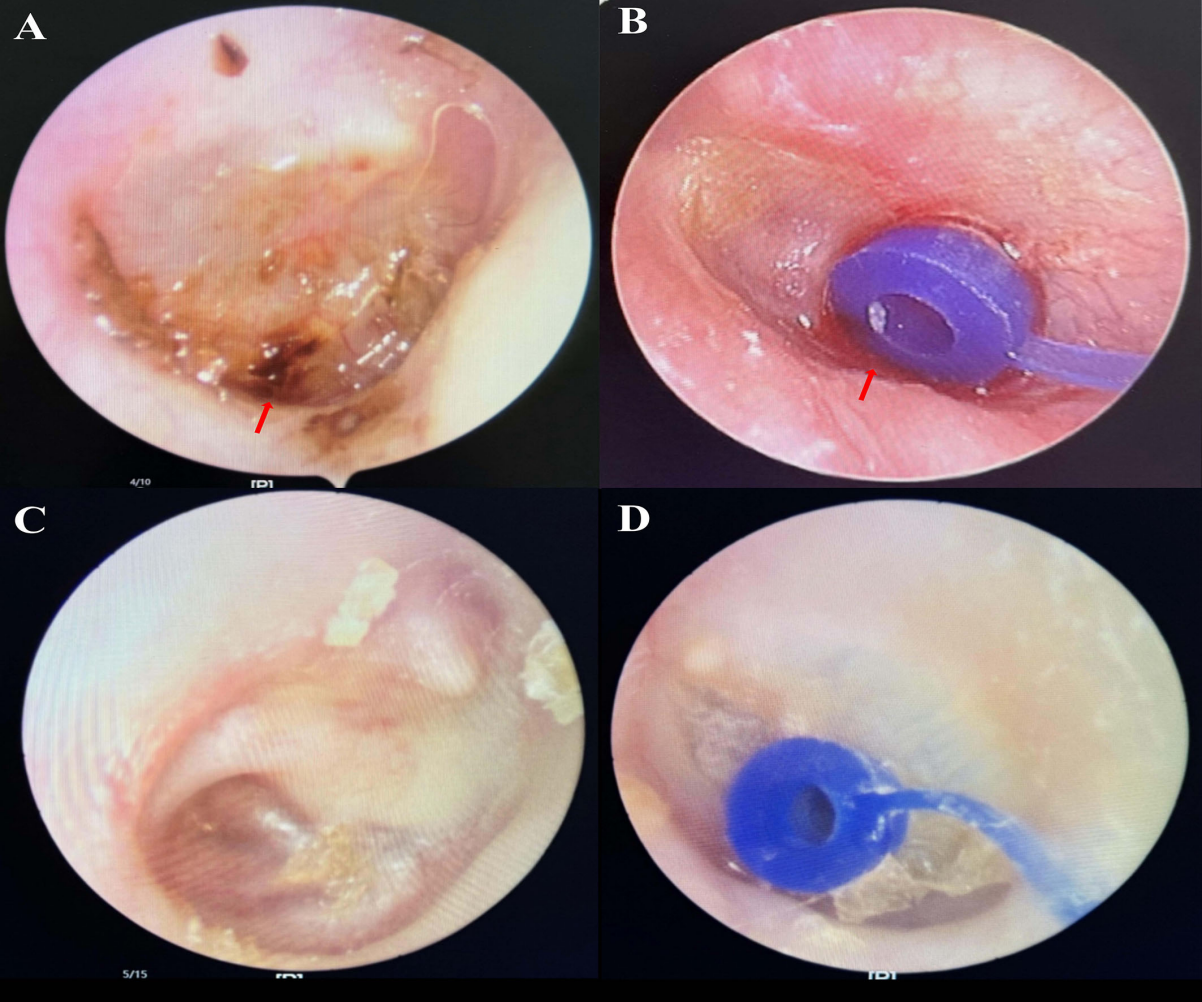
Otoendoscopy. **(A)** Right tympanic membrane hyperemia and edema. **(B)** Left sided tympanostomy tube insertion. **(C, D)** After treatment no discharge was observed in both ears, and the tympanic membranes showed no congestion.

**Figure 3:**
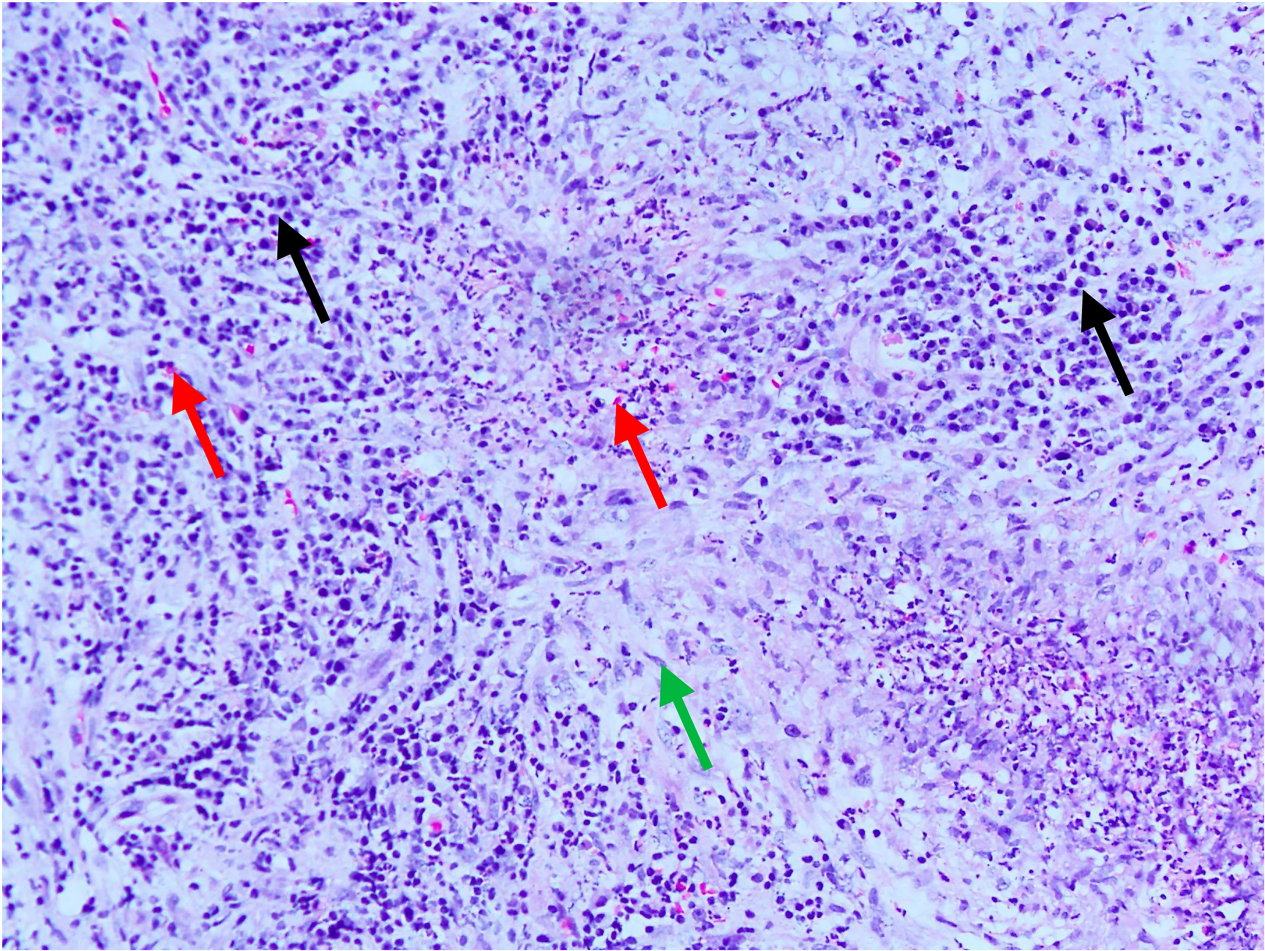
Postoperative pathological examination of surgical specimen (right mastoid). Black arrows: Inflammatory cells, predominantly plasma cells. Green arrows: Fibroblast proliferation. Red arrows: Mild congestion of a small number of capillaries.

On August 19, 2024, the patient was referred to the ENT Department of our hospital, where she was diagnosed with right-sided acute mastoiditis. After perfecting the otoendoscopy (**Figure 2A**), she underwent right-sided canal-wall-up mastoidectomy and exploratory tympanotomy. Then the postoperative pathological examination of surgical specimen revealed (right mastoid) a large number of inflammatory cells, predominantly plasma cells, are observed. Fibroblast proliferation is evident in the stroma, along with mild congestion of a small number of capillaries. No distinct nodular structures composed of epithelioid cells and multinucleated giant cells are apparent, suggesting the absence of granuloma formation (**Figure 3**). However, right sided otorrhea persisted postoperatively, and she developed an afternoon low-grade fever (maximum temperature [T_max_] 37.5 °C).

By September 2024, her condition progressed, with new-onset left-sided hearing loss, tinnitus, and otorrhea. The ENT department performed left sided tympanostomy tube insertion (**Figure 2B**) and administered another course of broad-spectrum cephalosporins and methylprednisolone (40 mg/day for 3 days), but symptoms remained unresolved.

On October 1, 2024, the patient’s bilateral hearing loss worsened suddenly. An outpatient ANCA panel was ordered, which showed a PR3-ANCA markedly elevated (200 RU/ml). She also reported a 5kg weight loss over the preceding 2 months and occasional productive cough, but denied other systemic symptoms including nasal congestion, epistaxis, crusting, hematuria, arthralgia, skin rash, oral ulcers and ocular discomfort.

### Admission evaluation

#### Vital signs and general status

Vital signs were stable with a temperature of 37.5 °C, and the patient was alert and oriented.

#### Physical examination

Purulent discharge was noted in both external auditory canals; the nasal cavity was clear. No skin rashes, hemorrhages or swollen lymph nodes were observed. Cardiac auscultation revealed a regular rhythm without murmurs; moist rales were audible at the left lung base. Abdominal examination and neurological assessment were unremarkable.

#### Auxiliary examinations

Laboratory tests showed normal levels of white blood cells, lymphocytes, and procalcitonin (PCT). The erythrocyte sedimentation rate (ESR; 30 mm/h) and high-sensitivity C-reactive protein (hs-CRP; 14.9 mg/L) were elevated. And the proteinase 3 antineutrophil cytoplasmic antibodies (PR3-ANCA; 200 RU/mL) were strongly positive, which suggested an active autoimmune process. T-SPOT and purified protein derivative (PPD) tests were both negative. Urinalysis, renal function tests, complement levels and antinuclear antibody (ANA) panel were all within normal ranges. In addition, chest computed tomography (CT) showed multiple bilateral solid pulmonary nodules with smooth margins, the largest located in the right lower lobe (27×15mm) (**Figures 1C, D**).

### Clinical diagnosis

Based on the clinic and laboratory findings, including strong PR3-ANCA positivity, bilateral pulmonary nodules on chest CT and refractory symptoms to antibiotic therapy, the patient was diagnosed with Granulomatosis with Polyangiitis (GPA) affecting the ears and lungs.

### Treatment and outcome

#### Treatment plan

The patient was initiated on immunosuppressive therapy, consisting of: (1) Glucocorticoid pulse therapy: Intravenous methylprednisolone 200mg daily for 3days, followed by an oral methylprednisolone taper starting at 40 mg daily; (2) Rituximab induction: Intravenous Rituximab 500 mg (equivalent to 375 mg/m²) weekly for a planned 4-week course.

#### Treatment outcome

Clinical improvement was observed as early as the 3rd day of glucocorticoid therapy: bilateral otorrhea resolved completely, and tinnitus and otalgia significantly improved. After discharge, the patient completed the planned rituximab treatment. Meanwhile, the oral methylprednisolone dosage was gradually tapered, and by the fourth week of therapy, it had been reduced to 16 mg per day.

#### Follow-up

Month Follow-Up: The patient reported marked improvement in hearing, with no recurrence of otorrhea. A repeat chest CT showed significant reduction in the size of bilateral pulmonary nodules, with complete resolution of some smaller lesions (**Figures 1E, F**) and the inflammatory markers (ESR, CRP) returned to normal ranges.

Mid-Term Follow-Up: The patient remained clinically stable with persistent remission of otologic and respiratory symptoms. On May 21, 2025 (7 months after the initial diagnosis and completion of 4 initial RTX doses), she presented for a follow-up visit. Compared with her first visit to Rheumatology Immunology Department, the hearing test shows significant improvement (**Supplementary Figures 1B, C**). Otoendoscopy shows no discharge was observed in both ears, and the tympanic membranes showed no congestion (**Figures 2C, D**). And the PR3-ANCA test was negative. After evaluating her disease activity (normal inflammatory markers, no new symptoms, and stable chest CT), the clinical team administered the 5th dose of intravenous RTX (500 mg) as part of her maintenance therapy to prevent disease relapse.

**Figure 4:**
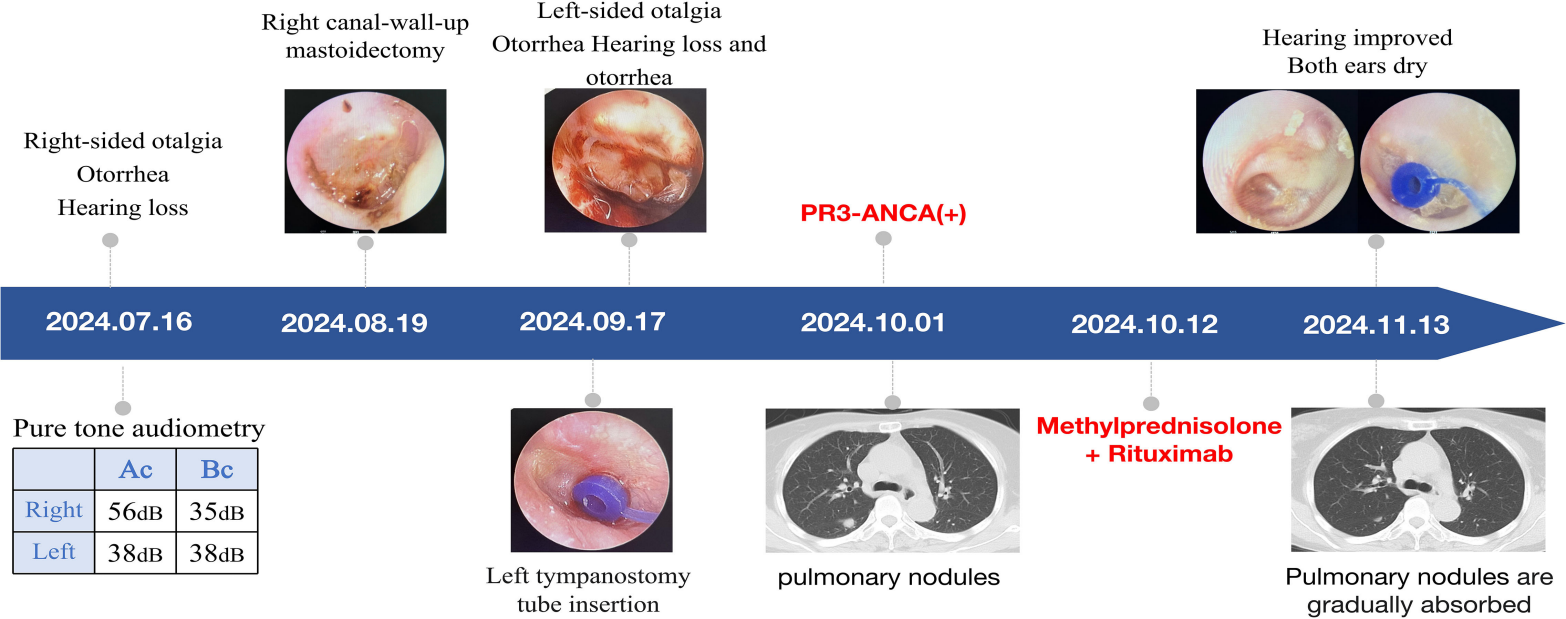
Timeline.

Long-Term Follow-Up: As of August 2025 (10 months after diagnosis and 3 months after the first RTX maintenance induction therapy), The patient remains in clinical remission (defined by persistent resolution of otologic symptoms, normal inflammatory markers, and stable chest CT). Her hearing has recovered to near-normal levels (**Supplementary Figure 1B**), with no evidence of disease relapse or adverse events related to the RTX. To clearly demonstrate the patient’s disease progression and treatment response, the key time points, examination results, and intervention measures are summarized in **Figure 4**. And our reporting of this study conforms to the Case Report (CARE) guidelines (7).

## Discussion

This case describes a patient with PR3-ANCA positive GPA who initially presented with refractory, destructive mastoiditis, highlighting the clinical challenge of this rare manifestation. This case is rare due to prolonged isolated otologic symptoms (3 months) without sinonasal or renal involvement, both of which are uncommon in GPA; consequently, this led to misdiagnosis as typical bacterial mastoiditis, resulting in ineffective antibiotic therapy and unnecessary surgical intervention.

Notably, this case underscores the importance of including GPA in the differential diagnosis of refractory otologic disease, and a key clinical takeaway given the potential consequences of diagnostic delay. While otologic involvement in GPA (e.g., otitis media) has been reported in 19%–40% of cases (4), this otologic involvement is classically considered secondary to nasopharyngeal inflammation and Eustachian tube dysfunction (5). Whereas this patient presented with isolated mastoiditis as the initial manifestation, accompanied by mixed hearing loss. GPA only rarely initially manifests with exclusive otologic involvement (8).

This case further reveals two critical diagnostic pitfalls in identifying GPA presenting as refractory otologic disease, which directly contributed to the delayed diagnosis observed: (1) Ineffectiveness of Surgical Intervention in Active Disease: The patient underwent mastoidectomy after failing antibiotic therapy; however, her symptoms not only persisted but also progressed to involve the contralateral ear. This clinical course aligns with evidence from prior studies, which suggest that surgical trauma during the active phase of ANCA-associated vasculitis (AAV) may paradoxically worsen local lesions (9)—a key consideration often overlooked in the management of refractory otologic disease; (2) Confounding Role of Non-Specific Pathology Report: The surgical pathology of the patient’s mastoid specimen showed only “inflammatory granulation tissue” (**Figure 3**). While the histopathological gold standard for GPA is “necrotizing granulomatous vasculitis”, obtaining this classic pathological finding from small, localized otologic biopsy specimens is extremely challenging. A seminal study by Takagi et al. specifically noted that ear-derived biopsies frequently exhibit non-specific inflammation or granulation tissue rather than the classic GPA pathology (4). Thus, in the context of destructive otologic disease refractory to conventional antibiotic and surgical treatments, a “non-specific” pathological report should be interpreted as a strong clue supporting (rather than excluding) a potential GPA diagnosis rather than dismissing the possibility of systemic vasculitis.

In recent years, the Japan Otological Society has proposed a distinct subtype termed “Otitis Media with ANCA-Associated Vasculitis (OMAAV)” (10). A nationwide analysis of 235 OMAAV patients demonstrated a predominance of myeloperoxidase (MPO)-ANCA positivity (about 60%), with this subtype often associated with facial nerve palsy (about 40%) and hypertrophic pachymeningitis (about 30%) (11). Notably, this contrasts with the current case, which was PR3-ANCA positive and lacked these common OMAAV-associated complications further highlighting the phenotypic heterogeneity of AAV related otologic disease.

Our patient’s phenotype differs significantly from that of typical OMAAV, with key distinctions in three domains: (1) Serological Profile: The patient was strongly PR3-ANCA positive, in contrast to the predominance of MPO-ANCA positivity in typical OMAAV. Importantly, PR3-ANCA is well-documented to be associated with the “classic” GPA phenotype—characterized by a higher incidence of pulmonary granulomas (e.g., the nodules observed in this patient) and an increased risk of disease relapse compared to MPO-ANCA positive AAV. (2) Clinical Symptoms: Unlike typical OMAAV (which often presents with facial nerve palsy and hypertrophic pachymeningitis-related severe headaches), the patient had no facial palsy or severe headache throughout her clinical course. (3) Systemic Involvement: The presence of multiple bilateral pulmonary nodules confirmed the systemic nature of her disease, which contrasts with the more localized otologic focus often seen in OMAAV.

Thus, this case does not represent a localized OMAAV subtype but rather a classic PR3-ANCA driven systemic GPA. Its uniqueness lies solely in the initial presentation of isolated destructive mastoiditis, which an atypical manifestation of otherwise classic systemic GPA.

Moreover, we excluded fungal mastoiditis, malignancy, microscopic polyangitis (MPA), and eosinophilic granulomatosis with polyangitis (EGPA) through histopathological and microbiological evaluation prior to diagnosing granulomatosis with polyangitis (GPA); however, confirmatory histopathological evidence of renal involvement could not be obtained due to patient refusal of renal biopsy.

Although the patient does not meet the diagnostic criteria for GPA established in 1990, a diagnosis of GPA was ultimately made based on the clinical manifestations, laboratory findings, and imaging results. We retrospectively consult the 2022 American College of Rheumatology/European Alliance of Associations for Rheumatology (ACR/EULAR) classification criteria for GPA (12) and the ACR/EULAR 2017 provisional classification criteria for GPA (13), our patient meets the aforementioned two classification criteria, which appears to corroborate our diagnosis.

A critical turning point in this patient’s management was the initiation of targeted immunosuppressive therapy. Prior to this, she had shown refractoriness to conventional treatments: broad-spectrum antibiotics, surgical intervention (right-sided mastoidectomy and left-sided tympanostomy tube insertion), and even moderate-dose oral glucocorticoids (methylprednisolone 40 mg/day for 3 days). In contrast, her otologic symptoms (otorrhea, tinnitus, otalgia) resolved rapidly upon initiating high-dose methylprednisolone pulse therapy (200 mg/day intravenously for 3 days) combined with RTX.

The selection of RTX as part of the induction regimen is supported by high-quality evidence from evidence-based medicine. The landmark Randomized Trial of Aggressive vs. Standard Therapy in ANCA-Associated Vasculitis (RAVE trial) compared RTX with cyclophosphamide (CYC) for remission induction in AAV. This trial demonstrated two key findings: first, RTX was non-inferior to CYC in inducing clinical remission; second, RTX was superior to CYC in patients with relapsing AAV (14). Building on this evidence, the latest 2022 EULAR recommendations for the management of AAV explicitly endorse RTX combined with glucocorticoids as the first-line induction therapy for newly diagnosed GPA patients with organ- or life-threatening disease (15, 16). For our patient, an elderly female with confirmed pulmonary involvement, RTX was particularly appropriate. Beyond its established efficacy in inducing remission, RTX offers a more favorable long-term safety profile compared to CYC, including a lower risk of treatment-related toxicities such as hemorrhagic cystitis and gonadal damage, critical considerations in elderly patients.

## Conclusion

This case describes a rare presentation of PR3-ANCA positive GPA, where the disease initially manifested as isolated, refractory destructive mastoiditis, an atypical initial manifestation that contributed to a 3-months diagnostic delay. This report offers three core takeaways for clinicians managing refractory otologic disease: (1)A high degree of suspicion for GPA is essential when encountering “refractory mastoiditis” that fails to respond to conventional antibiotic therapy and surgical intervention. Such refractoriness should prompt clinicians to consider systemic vasculitides rather than limiting differential diagnoses to infectious etiologies; (2) Given the low diagnostic yield of classic GPA pathology from otologic biopsies, a pathological report documenting “non-specific inflammatory granulation tissue” should not exclude a GPA diagnosis; (3) ANCA serology (including PR3-ANCA and MPO-ANCA) is a highly effective screening tool for GPA. The 3-months diagnostic delay in this case underscores the importance of early ANCA testing in patients with refractory otologic disease—this is critical for preventing irreversible hearing loss and avoiding further organ damage.

## Data Availability

The raw data supporting the conclusions of this article will be made available by the authors, without undue reservation.
