# Supplementary figures and images for: Refractory mastoiditis as the initial manifestation of granulomatosis with polyangiitis:a case report and literature review
Source: Front Immunol. 2026 Jun 29;17:1753797. doi: 10.3389/fimmu.2026.1753797 (PMC13357505; doi:10.3389/fimmu.2026.1753797)

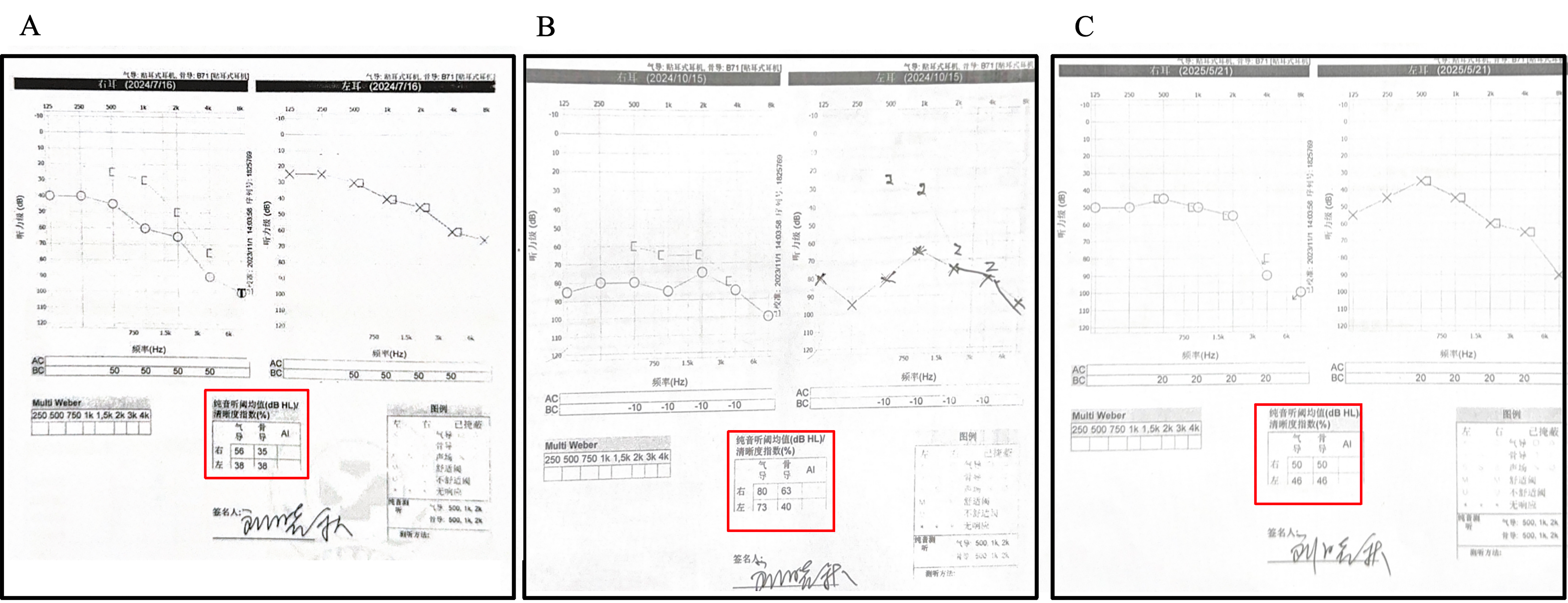

Supplement: Supplementary file 1 [file Image1.jpeg]
